# Dislocation between the Second and Third Cervical Vertebræ in a Horse, without Any Paralysis

**Published:** 1894-12

**Authors:** A. W. Clement

**Affiliations:** Baltimore


### DISLOCATION BETWEEN THE SECOND AND THIRD CERVICAL VERTEBRÆ IN A HORSE, WITHOUT ANY PARALYSIS.

By A. W. Clement, V.S., Baltimore.

A bay three-quarter thoroughbred gelding fell in jumping a fence in the hunting field, landing on his knees and at the same time striking his head and twisting his neck. The horse got up immediately and he was ridden home. The rider says that he persisted in carrying his head low but went along all right. He did not attempt to make him trot.

It was some three weeks after the accident occurred before I saw the case. The conditions then presented were as follows: The crown of the occiput was in a straight line from the withers or most prominent superior spinous processes of the dorsal vertebræ. There was a convex deviation from the straight line on the right side of about two inches, extending over the second and third cervical vertebræ and a corresponding concavity on the left side. The point of the nose and mouth was carried to the right of a straight line through the face about one and one-half inches. The head could be moved laterally on the neck about eighteen inches and up and down about as usual. The animal could turn his body to the left as well as ever but was somewhat awkward in turning to the right. The end of the third vertebræ could be distinctly felt jutting out beyond or to the side of the second vertebræ. The animal was given me for experimentation, and it was determined to operate, though with no hope of success, as adhesive inflammation had gone so far as to preclude any chance of reduction, even were such a thing possible at first. However, the animal was cast, chloroformed, and extreme extension with great pressure tried without any success. The second and third vertebræ were then completely exposed along the greater curvature of the dislocation. It was found there was fracture of the side of body of the vertebræ as well as dislocation, so that the pieces of bone could be pressed with the finger. It was then decided that any further efforts at operating would be useless and probably destroy the appearance of the specimen for anatomical purposes. I decided to close up the wound and allow the animal to live for a while for purposes of observation. This was done and the animal is now doing duty at light driving in the country near here. Later on the animal will be destroyed and the specimen exhibited to the medical profession. Several physicians have already seen the case and any who may be interested are quite welcome to examine the animal and to attend the autopsy, due notice of which, should the editor desire, will be given in this journal. *Maryland Medical Journal, Vol. XXXI. No. 17.*

